# A Machine-Learning-Based Prediction Method for Hypertension Outcomes Based on Medical Data

**DOI:** 10.3390/diagnostics9040178

**Published:** 2019-11-07

**Authors:** Wenbing Chang, Yinglai Liu, Yiyong Xiao, Xinglong Yuan, Xingxing Xu, Siyue Zhang, Shenghan Zhou

**Affiliations:** School of Reliability and Systems Engineering, Beihang University, Beijing 100191 China; changwenbing@buaa.edu.cn (W.C.); xuxx96@buaa.edu.cn (X.X.); zhang_sy@buaa.edu.cn (S.Z.)

**Keywords:** hypertension outcomes, feature selection, recursive feature elimination, classification algorithm, XGBoost, prediction

## Abstract

The outcomes of hypertension refer to the death or serious complications (such as myocardial infarction or stroke) that may occur in patients with hypertension. The outcomes of hypertension are very concerning for patients and doctors, and are ideally avoided. However, there is no satisfactory method for predicting the outcomes of hypertension. Therefore, this paper proposes a prediction method for outcomes based on physical examination indicators of hypertension patients. In this work, we divide the patients’ outcome prediction into two steps. The first step is to extract the key features from the patients’ many physical examination indicators. The second step is to use the key features extracted from the first step to predict the patients’ outcomes. To this end, we propose a model combining recursive feature elimination with a cross-validation method and classification algorithm. In the first step, we use the recursive feature elimination algorithm to rank the importance of all features, and then extract the optimal features subset using cross-validation. In the second step, we use four classification algorithms (support vector machine (SVM), C4.5 decision tree, random forest (RF), and extreme gradient boosting (XGBoost)) to accurately predict patient outcomes by using their optimal features subset. The selected model prediction performance evaluation metrics are accuracy, F1 measure, and area under receiver operating characteristic curve. The 10-fold cross-validation shows that C4.5, RF, and XGBoost can achieve very good prediction results with a small number of features, and the classifier after recursive feature elimination with cross-validation feature selection has better prediction performance. Among the four classifiers, XGBoost has the best prediction performance, and its accuracy, F1, and area under receiver operating characteristic curve (AUC) values are 94.36%, 0.875, and 0.927, respectively, using the optimal features subset. This article’s prediction of hypertension outcomes contributes to the in-depth study of hypertension complications and has strong practical significance.

## 1. Introduction

Hypertension is the most common chronic disease, and it is also the most important risk factor of cardiovascular and cerebrovascular diseases. Hypertension is the leading preventable cause of premature death worldwide. According to Mills et al., in 2010 there were approximately 1.33 billion people with hypertension worldwide, accounting for 19.3% of the world’s total population [1]. According to Kearney et al., by 2025 global hypertension patients will reach 1.56 billion [2]. Outcome of hypertension is a clinical concept that refers to the death or serious complications (myocardial infarction, stroke, etc.) that may occur in patients with hypertension. In clinics, the incidence of outcomes is not high, but once they occur they cause irreversible serious injury to patients.

Complications of hypertension refer to the diseases caused by hypertension. Studies have shown that hypertension is associated with a variety of adverse clinical symptoms, including cardiac complications, stroke, atherosclerosis, hypertensive kidney damage, and so on. These undesirable clinical features are the result of target organs damaged by hypertension [3,4]. Complications caused by hypertension pose a serious threat to patients’ health and life. Because about 50% of young and middle-aged hypertensive patients are asymptomatic, it is not easy for them to detect the threat of complications. Studies show that only 35.5% of patients have a good understanding of hypertension complications and related treatments [5]. Hypertensive complications are an important cause of death in hypertensive patients and impose a huge burden on families and the whole society [6]. 

In 2014, to determine the risk factors for complications of hypertension among patients attending the medical Out-Patient Department (OPD) of Sree Mookambika Institute of Medical Sciences (SMIMS), Devadason [7] obtained 100 hypertensive patients. 100 non-hypertensive patients from the same OPD were used as control group. Through analysis and comparison, family history of hypertension (Odds Ratio = 2.614, *p* = 0.002) and obesity (Odds Ratio = 1.833, *p* = 0.040) were determined as the main risk factors for hypertension. In his research, the number of factors affecting complications was not sufficient. In 2014, Wonji Lee et al. [8] used the classification algorithm to predict hypertension complications. This is the only research on prediction of hypertension complications in the literature so far. They used the sample national healthcare database established by Korean National Health Insurance Corporation for this research. The factors they chose included socio-demographic variables, medical treatment records, health check-up indices, behavior variables, and family history. Finally, they selected 27 influencing factors. The data set contained a total of 10,814 hypertensive patients, including 1739 patients with complications. Patients with complications accounted for 17% of the total number. They used three algorithms of logistic regression, linear discriminant analysis, and classification and regression trees to predict complications. After five-fold cross-validation, the accuracy rates of the three methods on the test set were not more than 60%. The three methods they proposed for predicting complications were less effective, and were obviously unsatisfactory for the binary task. These methods do not make much sense in practical applications (prediction of complications).

Various machine learning techniques have been widely used in medicine, such as disease prediction, disease classification, and medical image recognition techniques. Ospina et al. proposed a random forest tissue complication probability model (RF-NTCP) to predict late rectal toxicity after radiotherapy for prostate cancer. The area under the receiving operating characteristic curve (AUC) of this model is between 0.62 and 0.69. He used random forests to predict breast cancer survival, which are more accurate than traditional logistic regression methods [9]. Su used decision tree and serum test data to predict whether an individual had gastric cancer. Experiments showed that the accuracy of this method was 86.45% [10]. Hassan et al. proposed a hill-climbing feature selection algorithm that combines machine learning techniques (multi-layer perceptron, support vector machines, C4.5, classification and regression trees, and random forests) to more accurately analyze and predict pregnancy after IVF treatment. They put forward an effective method to identify the impact factors and the prediction effect was very good [11]. Austin used a machine learning method to classify heart failure subtypes [12]. Man et al. proposed an optimal weight learning machine and applied it to handwritten image recognition [13]. 

Medical research on hypertension has always been a great concern. Many hospitals and research institutes collect medical data of hypertension patients through follow-up, establish electronic cases and medical databases, and accumulate a large amount of medical data. This provides data support for predicting outcomes of hypertension and identifying key influencing factors using machine learning technology. It is very effective to use machine learning models to automatically identify the significant factors of outcomes and establish an effective prediction model. The knowledge gained from these machine learning models will help doctors make decisions, make treatment plans, and give patients effective advice. Ye et al. used the statewide electronic health record to predict the risk of hypertension within a year using the extreme gradient boosting (XGBoost) method [14]. Park used different machine learning methods to predict high-risk vascular disease in patients with hypertension [15].

For hypertensive patients and doctors, whether the hypertensive outcomes occur are the events that they are most concerned about. Since the impact of outcomes on patients’ life and health is often irreversible, the incidence of outcomes must be reduced. To achieve effective prevention, we must first effectively predict the outcomes. Only by effectively predicting the outcomes can doctors provide targeted treatment interventions for patients and control the patient’s condition, so as to effectively reduce the occurrence of outcomes that patients are most concerned about. Despite the enormous harm caused by hypertension outcomes, there are few studies on how to predict hypertension outcomes (death or complications). In this paper, some classical machine learning algorithms were used to find the influencing factors of hypertension outcomes and predict the outcomes effectively. These machine learning models can automatically help doctors identify key factors in a large number of medical indicators. At the same time, these models give suggestions on whether hypertension patients will have outcomes and help doctors make informative decisions. The key factors and potential knowledge identified by these machine learning models may play a positive role in medicine, better promoting the research and treatment of hypertension outcomes. Regarding the research object and the characteristics of the data set, this paper selects the following four machine learning techniques: support vector machine (SVM), C4.5 decision tree, random forest, and XGBoost. SVM can achieve better generalization ability in small sample classification tasks, and has been widely used in medical fields. C4.5 decision tree also achieves good results in classification tasks, and has good interpretability. Random forest and XGBoost are two typical ensemble learning algorithms. Random forest has been widely used in medical fields. XGBoost is a relatively new method, but it has achieved excellent results in many classification tasks. In addition, we used recursive feature elimination with cross-validation (RFECV) method for feature selection. This method combined with a classifier can identify the most influential factors and improve the prediction performance.

Support vector machine (SVM) was first proposed by Corinna, Cortes, and Vapnik in 1995 [16]. It has many unique advantages in solving small-sample, nonlinear, and high-dimensional pattern recognition. Given a sample set, the basic idea of classification learning is to find a partition hyperplane in the sample space based on the training set. This partition hyperplane can separate different categories of samples. There may be many hyperplanes with which to divide the training set. SVM aims to find a hyperplane so that different classes of points in the training sample set fall on both sides of the hyperplane, and the blank area on both sides of the hyperplane is required to reach the maximum. For two-dimensional linear separable data, SVM can theoretically achieve the optimal classification. When it is extended to high-dimensional space, the optimal classification line is called the optimal hyperplane [17]. SVM has a strong theoretical foundation and can ensure that the extremum solution is the global optimal solution rather than the local minimum, which means that the SVM method has good generalization ability for unknown samples. Because of these advantages, SVM can be well-applied to pattern recognition, time series prediction, and regression estimation, among others. It is also widely used in many fields, such as handwritten character recognition, text classification, image classification, and recognition [18,19,20,21,22]. 

Decision tree is a common machine learning algorithm, which uses a “tree structure” to make decisions. Decision tree is easy to understand because of its simple hierarchy and processing mechanisms. Generally, a decision tree contains one root node, several internal nodes, and several leaf nodes. Leaf nodes correspond to the decision results, and other nodes correspond to an attribute test. The samples contained in each node are divided into sub-nodes according to the results of attribute test, and the root node contains the entire sample set. The path from the root node to the final leaf node corresponds to a decision test sequence. The process of decision tree training follows a simple and intuitive “divide and conquer” strategy, which is a recursive process. The purpose of decision tree learning is to produce a decision tree with strong generalization ability [23].

For a data set, a decision tree is generated in a variety of forms. The key to decision tree learning is how to select the optimal partitioning attributes. Shannon entropy is a good way to divide attributes. Based on this, Hssina invented the Iterative Dichotmizer 3 (ID3) and C4.5 decision tree algorithms. ID3 uses information gain as a measure of feature selection, while C4.5 uses information gain ratio as a measure of feature selection. C4.5 inherits the advantages of ID3, and the prediction effect is usually better than ID3 [24]. Based on different attribute partitioning principles, a decision tree has many branches. Studies have shown that a C4.5 decision tree usually achieves better classification results than other decision trees [25].

Random forest (RF) is an extension of bagging method [26], a typical ensemble learning method. The principle of bagging method is as follows: Given a data set containing m samples, one sample is randomly selected and put into the sample set, and then the sample is put back into the initial data set, so that the sample may still be selected at the next sampling time. In this way, after m random sampling operations, we get a sample set with m samples. Some samples in the initial training set appear many times in the resampling set, and some never appear. T samples containing m training samples are selected, then a basic learner is trained based on each sample set, and then these basic learners are combined. Bagging usually uses a simple voting method for tasks. The base learner of RF is a decision tree, and random attribute selection is introduced in the training process of the decision tree. RF is simple, understandable, computationally inexpensive, and has achieved powerful performance in many real-world tasks, known as “methods representing the level of ensemble learning technology”. RF has been applied in gene selection, remote sensing classification, image recognition, and disease prediction, among others, and has achieved good results [27,28,29].

Extreme gradient boosting (XGBoost) is a new gradient boosting ensemble learning method. It is a C++ implementation of Tianqi’s gradient boosting tree algorithm. It implements a machine learning algorithm under the framework of gradient boosting, and has high efficiency, flexibility, and portability [30]. Tree boosting is a highly effective and widely used machine learning method that belongs to boosting ensemble learning [31]. Boosting is a family of algorithms that can turn weak learners into strong learners. Tree boosting (gradient tree boosting is also known as gradient boosting machine (GBM) or gradient boosted decision tree (GBDT)) has been shown to give state-of-the-art results on many standard classification benchmarks [32]. 

The GBDT algorithm only uses the first derivative. The value of the current *n*th tree is related to the residual of the first n−1 trees, and it is difficult to achieve. XGBoost takes advantage of the second-order Taylor expansion of the loss function, and adds a regularization term to balance the complexity of the model and the decline of the loss function. It seeks the optimal solution as a whole and avoids overfitting to some extent. XGboot can automatically implement gradient tree boosting algorithms in parallel by using a multi-thread CPU, which makes the algorithms run faster and improves the algorithm precision.

Suppose the model has *t* decision trees because XGBoost is a boosting algorithm based on residuals

(1) $${\hat{y}}_{i}{}^{\left(t\right)}=\sum_{k=1}^{t}f_{k}\left(x_{i}\right)={\hat{y}}_{i}{}^{\left(t-1\right)}+f_{t}\left(x_{i}\right),f_{k}\in F,i\in n$$

where ${\hat{y}}_{i}{}^{\left(t\right)}$ represents the predicted value of the sample *i*, which is the value obtained by adding the predicted values of *t* decision trees; *n* is the total number of samples, subscript *i* represents the *i*-th sample, *f_t_* is the *t*-th regression tree, and *F* is the collection space of all trees.

The loss function is:

(2) $$L^{\left(t\right)}=\sum_{i=1}^{n}l\left(y_{i},{\hat{y}}_{i}{}^{\left(t\right)}\right)+\sum_{k=1}^{t}\Omega \left(f_{k}\right)$$

where *l* represents the degree of deviation between predicted value ${\hat{y}}_{i}{}^{\left(t\right)}$ and true value $y_{i}$; the second part of the formula (2) represents the sum of complexity of each tree, and $\Omega \left(f_{k}\right)=\gamma *T+\frac{1}{2}\lambda {\left\|w\right\|}^{2}$, *T* is the number of leaf nodes, γ is the weight of leaf nodes, and λ and ω are regular coefficients.

Combining Equations (1) and (2) with the Taylor expansion of loss function, Equation (3) is obtained.

(3) $$\begin{array}{l} L^{\left(t\right)}=\sum_{i=1}^{n}l[y_{i},{\hat{y}}_{i}{}^{\left(t-1\right)}+f_{i}\left(x_{i}\right)]+\Omega \left(f_{t}\right)+\sum_{k=1}^{t-1}\Omega \left(f_{k}\right)=\\ \sum_{i=1}^{n}\left[l\left(y_{i},{\hat{y}}_{i}{}^{\left(t-1\right)}\right)+g_{i}f_{t}\left(x_{i}\right)+\frac{1}{2}h_{i}f_{{}_{t}}^{2}\left(x_{i}\right)\right]+\Omega \left(f_{t}\right)+\sum_{k=1}^{t-1}\Omega \left(f_{k}\right)\\ \sum_{i=1}^{n}\left[g_{i}f_{t}\left(x_{i}\right)+\frac{1}{2}h_{i}f_{{}_{t}}^{2}\left(x_{i}\right)\right]+\gamma T+\frac{1}{2}\lambda \omega_{j}^{2}+C \end{array}$$

where *g_i_* is the first derivative, *h_i_* is the second derivative, and *C* is the constant.

(4) $$g_{i}=\partial_{{\hat{y}}_{i}{}^{\left[t-1\right]}}l\left(y_{i},{\hat{y}}_{i}{}^{t-1}\right)$$

(5) $$h_{i}=\partial_{{}_{{\hat{y}}_{i}{}^{\left[t-1\right]}}}^{2}l\left(y_{i},{\hat{y}}_{i}{}^{t-1}\right)$$

(6) $$C=\sum_{i}^{n}l\left(y_{i},y_{i}^{\left[t-1\right]}\right)+\sum_{k=1}^{t-1}\Omega \left(f_{k}\right)$$

Definition $I_{j}=\{i|q\left(x_{i}\right)=j\}$ represents a sample set of leaf nodes *j*. After the constant term is removed from Equation (3), and the derivative is 0, the optimal solution $\omega_{j}^{*}$ can be obtained.

(7) $$\omega_{j}^{*}=-\frac{G_{j}}{H_{j}+\lambda }$$

(8) $$G_{j}=\sum_{i\in I_{j}}g_{i}$$

(9) $$H_{j}=\sum_{i=I_{j}}h_{i}$$

Bringing the optimal solution $\omega_{j}^{*}$ into Equation (3), we get Equation (10):

(10) $$L^{\left(t\right)}=-\frac{1}{2}\sum_{j=1}^{T}\frac{G_{j}^{2}}{H_{j}+\lambda }+\lambda T+C$$

XGBoost uses a greedy algorithm to segment existing nodes each time, in comparison to GBDT using partitioning criteria to minimize mean square deviation. Suppose *I_L_* and *I_R_* are the set of left and right nodes after segmentation, *I* = *IL* ∪ *IR*, then the information gain after segmentation is:

(11) $$L_{\left(split\right)}=\mathrm{Gain}=\frac{1}{2}[\frac{G_{L}^{2}}{H_{L}+\lambda }+\frac{G_{R}^{2}}{H_{R}+\lambda }+\frac{{\left(G_{L}+G_{R}\right)}^{2}}{H_{L}+H_{R}+\lambda }]-\gamma$$

(12) $$G_{L}=\sum_{i\in I_{L}}g_{i}\;G_{R}=\sum_{i\in I_{R}}g_{i}\;H_{L}=\sum_{i\in I_{L}}h_{i}\;H_{R}=\sum_{i\in I_{R}}h_{i}$$

As can be seen from Equation (11), similar to ID3, C4.5, and Classification and Regression Tree (CART), XGBoost determines whether a node is splitting by subtracting the unsplit node score from the left and right splitting node scores. At the same time XGBoost considers the complexity of the model, adding the regular term *λ* to limit the growth of the tree. When the gain is less than *λ*, no node splitting is performed.

In this paper, there are several innovations in comparison with previous predictions of hypertension complications: (1) The study is aimed at the outcomes of hypertension, and the outcomes are complications that pose a major threat to human life. There has been no previous research on this subject. Accurate prediction of high blood pressure outcomes can help doctors make judgments, and doctors can perform preventive treatment on patients based on the results. (2) Previous studies have used all the characteristics of patients for prediction. The method in this paper can automatically select the key factors that cause the outcomes of hypertension, thus reducing the complexity of the prediction method. (3) The method proposed in this paper has higher prediction accuracy and better effect. (4) The result of this paper is the successful application of machine learning methods in the medical field. It has important practical significance for the in-depth study of serious complications of hypertension.

The other chapters of this paper are arranged below. The second chapter introduces the methods and processes, the third chapter is the analysis and discussion of the results, and the fourth chapter is the conclusion.

## 2. Materials and Methods

### 2.1. Recursive Feature Elimination with Cross-Validation (RFECV)

Some data sets have very high feature dimensions, and even dimensions that exceed the number of samples in the data set. An excessive number of features does not mean that the model is better; on the contrary, it will lead to inefficiency in the modeling process. The main reason is that there is a lot of redundant information and noise in high-dimensional data. Therefore, how to extract useful information from high-dimensional data plays an important role in subsequent modeling. Feature selection algorithm can effectively delete redundant data and noise data and select the most relevant feature variables, so it can effectively reduce the dimensions of data. At present, there are many advanced feature selection technologies, such as F-score [33], which have good performance and are widely used.

The recursive feature elimination (RFE) method for feature selection has attracted much attention due to its better robustness [34]. In recent years, RFE has been widely used in protein classification, gene selection, expression analysis, cancer diagnosis [35,36,37,38,39], and other biomedical fields. RFE is a greedy algorithm and the representative of the wrapper model algorithm. Its search starting point is a complete set, and the evaluation principle is the prediction accuracy of the classifier. At the end of the iteration, the most irrelevant feature is eliminated. The most irrelevant features are eventually eliminated, so the most relevant features are ranked at the top to sort the features. RFE generates some feature subsets according to the feature sorting table generated by the above evaluation criteria. RFE can be combined with different classifiers, such as SVM, random forest [40], and others. The steps of the RFE algorithm are as follows:

(1) Initialize feature set *F.*
(2) Select classifier *C.*
(3) Calculate the weight of each feature *f_i_* in *F* (the criterion is the accuracy of classifier prediction).
(4) Delete the minimum weight feature *f_j_* and update the *F*.
(5) Repeat steps 3 and 4 until *F* has only one feature left.
(6) Feature importance ranking.

As mentioned above, the method of combining RFE with a classifier can output a list *l_n_* (*n* indicates the number of features included in the list *l*) sorted by the importance of features from large to small. However, the performance of the classifier is largely influenced by the number of selected features. Therefore, on the basis of feature ranking, we need to determine the optimal number of features. Using the sorted list *l_n_*, we can get a set of feature subsets $F_{1}\subset F_{2}\subset \cdots \subset F_{n}$. *F*_1_ is composed of the first feature in *l_n_*, *F_2_* is composed of the top two features in *l_n_*, and so on, and *F_n_* represents the complete feature set. Next, we select a classifier (SVM, C4.5 decision tree, RF, or XGBoost), train these feature subsets in turn through cross-validation, and use the classification performance (accuracy or F1 measure) of the classifier as the criteria to evaluate these subsets, so as to find the optimal feature subset. For a features set *F* containing *n* features, the number of all its subsets is 2*^n^* − 1 (including empty sets). Compared with the exhaustive method, the number of subsets that RFECV needs to verify is only *n*. When *n* is large, the calculation of RFECV is much smaller than the exhaustive method.

RFECV cross-validates different combinations of features on the basis of RFE. By calculating the sum of the decision coefficients, the importance of different features to the score is finally obtained, and then the optimal feature combination is retained. The feature subset selected by RFECV is affected by two aspects: one is the classifier combined with RFECV, and the other is the performance evaluation criteria of the classifier. Selecting different classifiers or performance evaluation criteria, feature subsets are often not the same. The RFECV method used in this paper uses 10-fold cross-validation. Ten-fold cross-validation is a commonly used test method to test the performance of models. The idea is to divide the data set into ten parts, and take turns to use nine of them as training data and one of them as test data. Each test yields a correct rate, and the average of the correct rate of the 10 results is used as an estimate of the accuracy of the algorithm. This method is very suitable for data sets with small amounts of data, and is more powerful than the hold-out method in evaluating the generalization ability of the algorithm [41]. In this paper, we use this method to train and test the model. All the samples in the data set are randomly divided into 10 mutually exclusive subsets of a similar size. In each round of training, 9 subsets are selected in turn to form the training set, and the remaining 1 subset forms the test set. In each exclusive subset, the ratio between positive and negative cases should be similar to that of the data set. This method allows the model to be trained and tested 10 times, each time using a different set of training and testing. We take the average of the 10 test results as the final result.

### 2.2. Data Preprocessing

#### 2.2.1. Missing Value Processing

Simple deletion and filling are the main ways to deal with missing values. The simple deletion method involves deleting items with missing values. This method is simple and easy to implement. It is very effective when the sample has many missing values and the deleted samples account for a small proportion of the data set. However, this method has great limitations. It can reduce the sample size in exchange for complete information, but it will cause a lot of waste of resources, discarding a lot of hidden information in these samples. In the case of few samples in the data set, deleting a small number of samples is enough to seriously affect the objectivity of the data set information and the correctness of the results.

In view of the characteristics of our data set, we first delete many records whose features are missing values from the data set, because these records do not provide enough valuable knowledge. In this study, records missing more than 50% of the feature values are considered noise data and deleted from the data set. In addition, when more than 50% of the value in the feature column is missing, we also delete the feature because there is too much missing data to fill.

For other remaining missing values, we use the mean completer method to deal with them. Features are divided into numerical features and non-numeric features for processing separately. If the null value is numeric, the missing value is filled in according to the average value of the property in all other samples; if the null value is non-numeric, the highest frequency of occurrence of the feature in other objects is used to fill in the missing value according to the statistical mode principle.

#### 2.2.2. Data Normalization

Normalization is due to the different dimensions or dimension units in different feature indices. This causes the data with different attributes to be in different orders of magnitude. This may cause some indicators to be ignored and affect the results of the model. After normalization, all attributes of the original data are in the same order of magnitude for comprehensive comparison and evaluation.

The normalization method commonly used is *max*–*min* standardization. It is a linear transformation of raw data, so that the result is mapped to (0–1). The transformation function is as follows:

(13) $$x^{*}=(x-\min)/(\max-\min)$$

Among them, *max* is the maximum value of the sample data, and *min* is the minimum value of the sample data.

The anonymized data set of this study is from the hypertension database of a cardiovascular hospital in Beijing. The data was extracted from patients from various provinces in China, so the sample is diverse. Data collection began in September 2012 and was completed in August 2016. A total of 1357 cases were collected. The data set is divided into two parts. The first part is the physical examination data of the patients at admission, and the other part is the data investigating whether the patients who were followed up with by doctors had outcomes or not. The data set contains 132 physical examination indicators. These indicators are divided into the following categories: baseline data, limb blood pressure, ambulatory blood pressure, echocardiography, heart failure, blood routine, blood biochemistry, metabolism, and endocrine. 

Table 1 is the original feature. In Appendix A, Table A1 is an explanation of the abbreviation in Table 1. Table 2 shows the name, medical meaning, data type mean value standard deviation (Std.), and data distribution range of some indicators of the data set.

After data preprocessing, the final data set consisted of 374 samples, 95 of which had hypertension outcomes and the remaining 279 had no outcomes. The data set dimension ultimately has 84 dimensions. Table 3 shows a part of the processed data set.

### 2.3. Model Performance Metrics

The generalization ability of the classifier should be evaluated according to the generalization ability of the model. In order to evaluate the performance of classifiers, we adopt the following evaluation criteria: accuracy (*ACC*), F1, and *AUC*. These are commonly used evaluation criteria for classifier performance measurement.

(1) Accuracy (ACC): Generally, the percentage of misclassified samples from the total samples is called the “error rate”. That is, if there are *a* samples in *m* samples, the error rate is *E = a/m.* Correspondingly, the calculation method for accuracy is as follows:

(14) ACC=(1−a)/m

(2) F1 Measure: Although *ACC* is commonly used, it does not satisfy all requirements. For the binary classification problem, the samples can be divided into four cases, true positive (*TP*), false positive (*FP*), true negative (*TN*), and false negative (*FN*), according to the combination of their real classes and classifier predicted classes. The confusion matrix of division results is shown in Table 4.

The following describes two metrics, precision (*P*) and recall (*R*).

(15) $$P=\frac{TP}{TP+FP}$$

(16) $$R=\frac{TP}{TP+FN}$$

Precision indicates that the percentage of real positive samples accounts for the number of samples predicted to be positive samples. Recall indicates that the percentage of samples predicted to be positive samples accounts for the number of real positive samples. The higher the *R* ratio, the smaller the *FN*, the less the missing samples, the more complete the search. F1 measure is defined based on the harmonic mean of precision and recall. It is a comprehensive consideration of precision and recall. The formula is as follows:

(17) $$F_{1}=\frac{2*P*R}{P+R}$$

(3) Area under ROC (AUC)

*AUC* is often used in biomedical works [42,43]. It is defined as the area under the receiver operating curve (*ROC*). *ROC* is a comprehensive indicator reflecting continuous variables of sensitivity and specificity. Each point on the *ROC* curve reflects the sensitivity to the same signal stimulus. The value of *AUC* will not be greater than 1, and since the *ROC* curve is generally above the line *y = x*, the range of *AUC* is generally between 0.5 and 1. The criteria for *AUC* to judge the performance of the classifier (predictive model) is:

If *AUC* = 1, the classifier is perfect, and with this predictive model, there is at least one threshold that yields a perfect prediction.

If 0.5 < *AUC* < 1, the classifier is better than random guessing. If the classifier properly sets the threshold, it can have predictive value.

If *AUC* = 0.5, the classifier follows the random guess and has no predictive value.

If *AUC* < 0.5, the classifier is worse than a random guess.

### 2.4. Diagnostic Process

**Figure 1:**
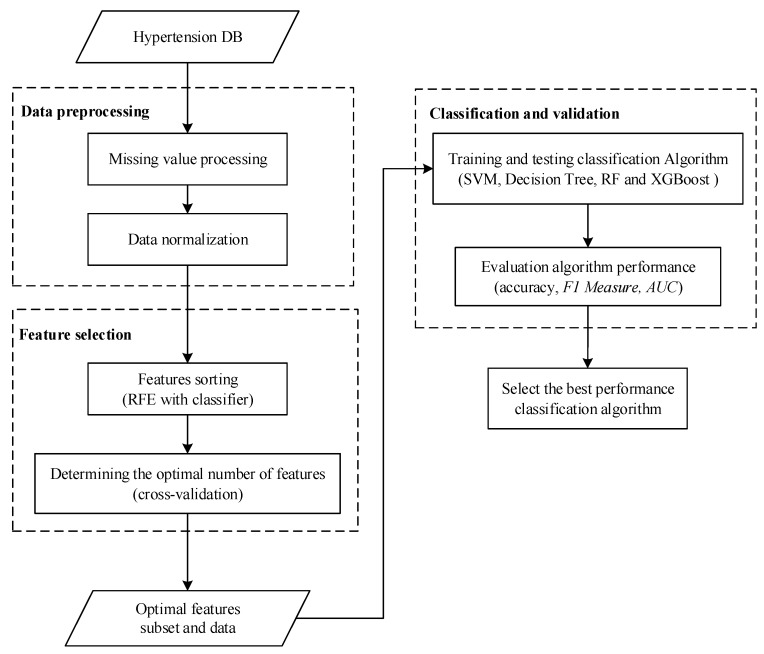
Predictive model construction process. Abbreviations: RF = random forest; AUC = area under receiver operating curve; RFE = recursive feature elimination; XGBoost = extreme gradient boosting; SVM = support vector machine; F1 Measure is calculated according to Equation (17).

The process diagram of the diagnosis is shown in Figure 1. Firstly, the data from patients with hypertension is obtained, the samples with missing values are processed, and then normalized. There are many physical examination indicators for patients. If all of them are brought into the model for training, it will increase the computational burden of the model. In most cases, these physical examination indicators will have a great correlation and reduce the accuracy of the results. For example, nighttime average systolic blood pressure and 24-h average systolic blood pressure have a strong correlation. Considering the computational burden and accuracy, dimensionality reduction of features is needed. Next, this paper chooses the RFE method, which uses cross-validation to preserve the best performance characteristics. Using this method, the optimal number of features under different conditions can be obtained according to different classifiers. Then, these features are extracted from the original sample to form a new sample set. Next, the new sample set is divided into a training set and test set. Four classifiers are used to construct the model, and then the test set is input into the model to get the results. The results are evaluated according to the accuracy, F1 measure, and *AUC* evaluation indicators, and the best performance model is finally selected.

## 3. Results and Discussion

According to the feature selection method proposed in this paper, the feature subset selected by RFECV is affected by two aspects: one is the classifier combined with RFECV, and the other is the performance evaluation criteria of the classifier. When selecting different classifiers or performance evaluation criteria, feature subsets are often not the same. Table 4 lists the number of optimal feature subsets for each classifier under three evaluation criteria.

From Table 5, it can be seen that the number of redundant features can be greatly reduced by the feature selection method proposed in this paper, which will help to save computational efficiency and improve the prediction effect of the model. It should be noted that because the number of samples in this paper is relatively small, there is no significant difference in the operating time between the four classifiers. Next, we use these feature subsets for training and testing. Under the F1 measure, the optimal number of features for SVM is 16, while under each criteria, the optimal number of features for C4.5 is 2. This result does not indicate that the lower the number of features needed, the better the performance. The next step is to evaluate the performance of the various methods in their respective best combinations.

The three features XGBoost selected under the F1 criteria are right lower extremity systolic blood pressure (RLEGSBP), left lower extremity diastolic blood pressure (LLEGDBP), and daytime mean diastolic blood pressure (DAYMDBP). 

In previous experiments, the optimal number of features required for each classifier was determined. All subsequent experiments were carried out with the optimal number of features. In the experiment, by changing the two parameters of XGBoost (i.e., the number of evaluators and the depth of the tree), we find the best combination of parameters for XGBoost, in which the number of evaluators is 50 and the depth of the tree is 5. Other models also use the best parameters selected after multiple tests.

**Figure 2:**
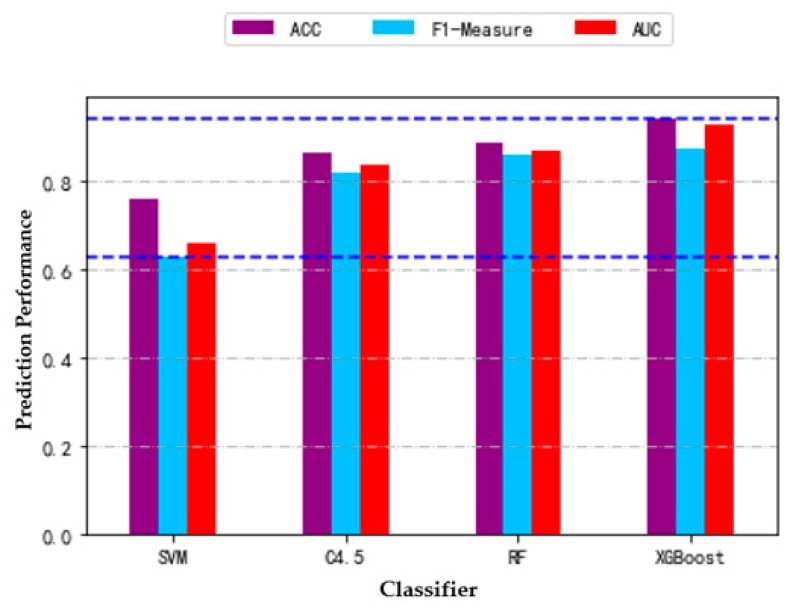
Prediction performance (accuracy, F1 measure, and *AUC*) of each classifier using their optimal features subset. (The blue dotted line represents the position of the maximum or minimum value).

The optimal feature subsets selected by RFECV method under *ACC*, F1 measure and *AUC* are used for modeling. Table 6 shows the performance of each classifier on different evaluation criteria. The results shown in the table are the average of the 10-fold cross-validation results. From the table, we can get the best results for the XGBoost classifier. Its accuracy is 94.36%, F1 measure is 0.875, and *AUC* is 0.927. The next is random forest. Its accuracy is 88.98%, and F1 measure and *AUC* are over 0.85. The third is the C4.5 decision tree, for which the accuracy is 86.03%, and F1 measure and *AUC* are over 0.8. The worst performance classifier is SVM. Its accuracy is 75.80%, while the F1 measure and *AUC* are not more than 0.7. Figure 2 shows more intuitively the performance scores of four classifiers under three performance metrics. It is obvious that XGBoost has excellent performance under three performance metrics, and SVM has the worst performance.

**Figure 3:**
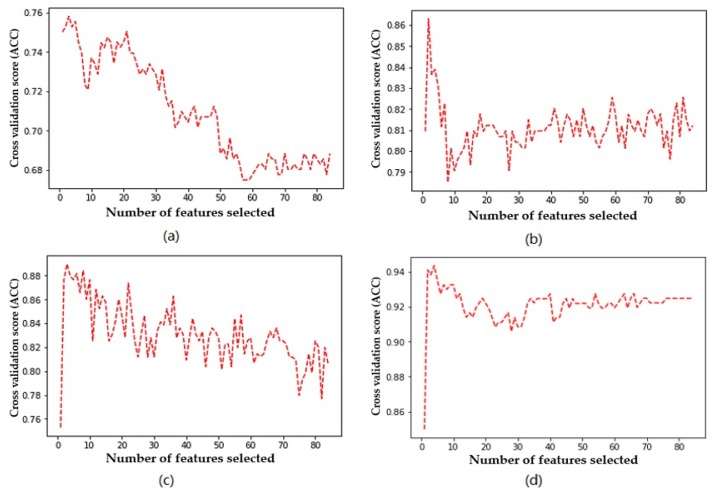
Relationship between accuracy and number of features used by the classifier: (**a**) SVM, (**b**) decision tree, (**c**) RF, (**d**) XGBoost.

**Figure 4:**
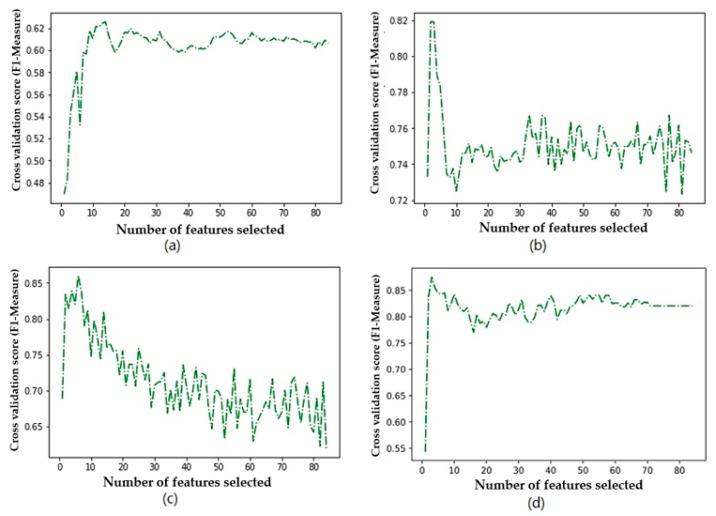
Relationship between F1 measure and number of features used by the classifier: (**a**) SVM, (**b**) decision tree, (**c**) RF, (**d**) XGBoost.

**Figure 5:**
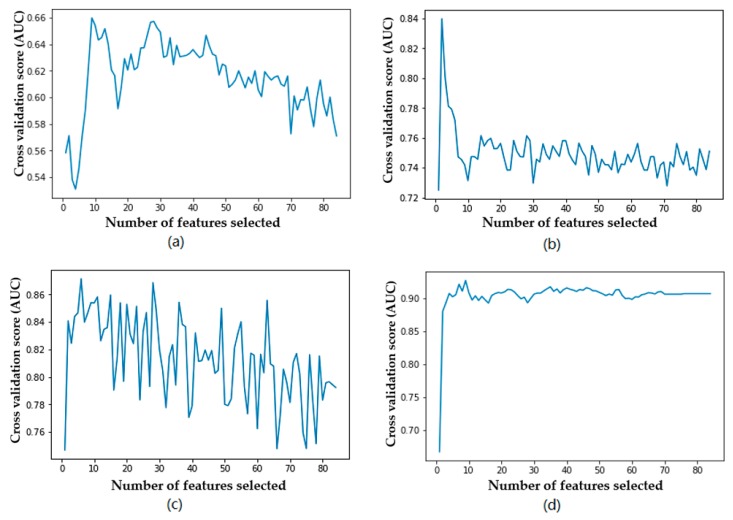
Relationship between *AUC* and number of features used by the classifier: (**a**) SVM, (**b**) decision tree, (**c**) RF, (**d**) XGBoost.

Figure 3 shows the trend of accuracy for the four classifiers using different numbers of features. Figure 4 shows the trend of the F1 measure for the four classifiers using different numbers of features. Figure 5 shows the trend of *AUC* for the four classifiers using different numbers of features. From Table 6 and Figure 3, we can find the highest accuracy of these four classifiers under different criteria. Cutting or adding other features will make the accuracy decrease. The accuracy of SVM, C4.5, and RF decreased significantly when the number of selected features exceeded the number of optimal subsets. This shows that adding features does not necessarily improve performance, and unrelated and redundant features may even degrade the performance of classifiers. The number of research object features in this paper reaches 84, and there must be many unrelated or redundant features. These features reduce the performance of the classifier and increase the amount of computation. A small number of important features selected by the REFCV method can achieve higher accuracy. In medical applications, fewer features mean that medical practitioners collect fewer physical indicators from patients. This is of practical significance for reducing medical costs and saving time for diagnosis and treatment. From Figure 3d, we can see that XGBoost used a small number of features to achieve high prediction accuracy, and with the increase in the number of features, accuracy remains stable. That is to say, XGBoost is insensitive to changes in the number of features, it is less affected by irrelevant and redundant features, and has high robustness. From Figure 4 and Figure 5 we can see the performance of the four classifiers under these two performance metrics is roughly the same as that under the accuracy. SVM, C4.5 decision tree, and RF achieve the best prediction results under their respective optimal subset of features, and the number of features used is very small. Then, with the increase of the number of features, the performance has a significant downward trend and volatility. The prediction performance of XGBoost is less affected by the number of features.

**Figure 6:**
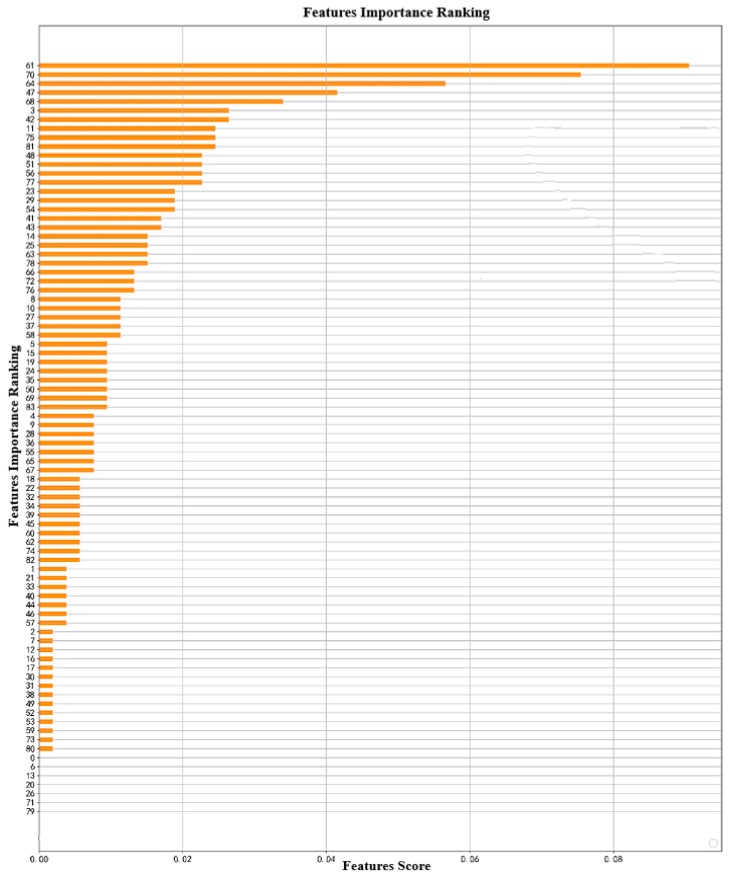
Feature weighting of XGBoost.

Figure 6 shows the ranking of importance of the XGBoost method. The number on the ordinate represents the number of features; for example, 61 represents the 62nd feature, because the numbers start from 0.

Through the experiment, the accuracy and *AUC* of the XGBoost–RFECV prediction model are 94.36% and 0.927, respectively. Using the proposed method, the number of features used by the classifier is very small, which reduces the difficulty of data collection and the cost of prediction. Generally speaking, the method is practical in helping doctors predict the outcome of patients with hypertension. 

**Figure 7:**
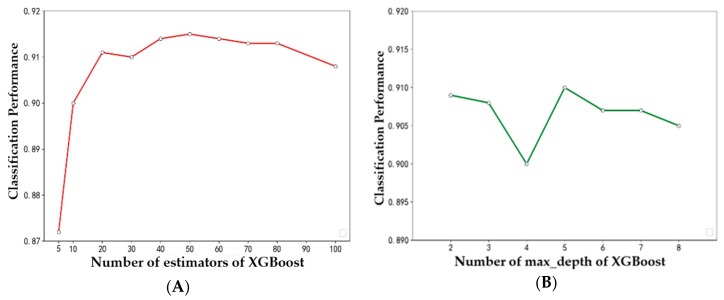
Classification performance of different (**A**) number of evaluators and (**B**) depths for XGBoost.

There are two important parameters in XGBoost: one is the depth of the tree and the other is the number of evaluators. In order to test the stability of XGBoost performance, the performance of XGBoost is calculated by changing the two parameters. Figure 7A shows the relationship between accuracy and the number of evaluators in XGBoost. With the increase of the number of evaluators, the computational accuracy is over 0.870 and stable at about 0.910. Figure 7B shows the relationship between the accuracy and the tree depth in XGBoost. With the increase of tree depth, the calculation accuracy is maintained at about 0.905. This shows that the XGBoost method has high stability. However, when the number of estimators exceeds 50 or the depth of transmission exceeds 5, the calculation accuracy of XGBoost decreases, which indicates that the sample size is relatively small. If the model constructed by XGBoost method is too complex, it will result in over-fitting.

## 4. Conclusions

Predicting the outcome of hypertensive patients is very meaningful research work. For this work, this paper proposes a method combining a classifier (SVM, C4.5 decision tree, RF, or XGBoost) with RFECV to accurately predict patient outcomes automatically. RFE method is used to assess the importance of physical examination indicators for hypertension outcomes. On this basis, cross-validation is used to find out the optimal feature subset to enhance the prediction performance of the classifier. Experiments show that the RFEVC method combined with C4.5, RF, and XGBoost can achieve better prediction performance. These three classifiers can achieve better predictive performance with only a small number of feature subsets. Among them, XGBoost has the highest prediction accuracy and good generalization ability, and is only slightly affected by the number of features. In addition, through RFECV we found that limb blood pressure and ambulatory blood pressure have important effects on the outcomes of hypertension.

The method proposed in this paper can effectively assist doctors to determine whether there will be outcomes in patients with hypertension. In this way, doctors can provide targeted interventions for patients with higher risk of outcomes and reduce the possibility of outcomes. The prediction model proposed in this paper requires only a small number of physical examination indicators. On the one hand, it reduces the cost of patients’ physical examinations and does not need many complicated physical examination items, thus improving the applicability; on the other hand, this method can be applied to telemedicine. Patients can measure their blood pressure by a simple self-test and transmit information to doctors through the network, so that doctors can evaluate the risk of patient outcomes. This will effectively reduce the cost and improve the efficiency of diagnosis and treatment.

Our future research will focus on the following issues. First, we will get a larger data set from more data sources to further test the generalization ability of our proposed method. Second, RFECV is a partial optimization algorithm, so we will improve the feature selection strategy to achieve better predictive effect. Third, we will verify whether other advanced classification algorithms that have better prediction performance, so as to provide doctors with more reliable, aided, automated decision-making tools. The more machine learning methods are applied to the medical field, the more accurate disease diagnosis will be.

## Figures and Tables

**Table 1:** Original feature.

No.	Name	No.	Name	No.	Name
	Baseline Data		Blood biochemical	57	RARMDBP
1	SEX	30	ALT	58	LARMSBP
2	AGE	31	AST	59	LARMDBP
3	HEIGHT	32	K	60	LLEGSBP
4	WEIGHT	33	Na	61	BAPWVR
5	BMI	34	Cl	62	RLEGSBP
6	HR	35	GLU	63	LLEGDBP
7	PULSE	36	CREA	64	RLEGDBP
8	RYSBPL	37	BUN	65	BAPWVL
9	RYDBPL	38	URIC	66	ABIR
10	HTBEGIN	39	HSCRP	67	ABIL
11	ZGSBP	40	TG		Dynamic blood pressure
12	ZGDBP	41	TC	68	MEANSBP
13	PSSBP1	42	HDLC	69	MEANDBP
14	PSDBP1	43	LDLC	70	HIGHSBP
	UCG cardiac vascular ultrasound		Thyroid function	71	DAYMDBP
15	AO	44	FT3	72	LOWSBP
16	LA	45	FT4	73	LOWDBP
17	IVSD	46	T3	74	DAYMSBP
18	LV	47	T4	75	HIGHDBP
19	EF	48	TSH	76	NIHTMSBP
20	LVPWd		Urine protein	77	NIHTMDBP
21	RVd	49	MAUCR		Breathing sleep
	blood routine	50	HUPRO	78	AHI
22	WBC		Blood sugar	79	APNEA
23	NEUT	51	HBLAC	80	HYPOPNEA
24	RBC		Inflammatory factor	81	SAO2
25	HB	52	ESR	82	MEANSAO2
26	PLT	53	CRP		Other
	Urine routine	54	NTPRO	83	HCY
27	UKET	55	ET	84	W_DISC_NOHPT
28	USG		Limb blood pressure		
29	USG1	56	RARMSBP		

**Table 2:** Description of partial hypertension examination indicators.

Attribute No.	Name	Description	Type	Value Range	Mean Value	Std.
1	Sex	Baseline data	Categorical	Male or female (1 or 0)	/	/
2	Age	Baseline data	Numeric	15–76	38.31	11.42
3	BMI	Body mass index	Numeric	10–50.93	27.28	4.27
4	PULSE	Pulse rate	Numeric	49–121	76.28	12.65
5	RYSBPL	Left arm systolic pressure	Numeric	95–230	151.90	22.67
6	FT3	One index of thyroid function	Numeric	0.74–7.3	3.19	0.47
7	SaO2	One index of respiratory sleep test	Numeric	55–96	84.13	6.54
8	meanSBP24h	24 h mean systolic blood pressure	Numeric	96–184	135.09	15.12
82	NIHTMDBP	Mean diastolic pressure at night	Numeric	48–131	82.23	12.57
83	NIHTMSBP	Mean systolic blood pressure at night	Numeric	87–192	128.20	17.03
84	W_DISC_NOH	Number of antihypertensive drugs at discharge	Categorical	0,1,2,3,4	/	/

**Table 3:** Processed data set.

	Item.	SEX	AGE	HEIGHT	WEIGHT	BMI	W_DISC_NOH ^1^
NO.							
**1**		1	36	168	65	23.03	3
**2**		1	55	178	105	33.13	3
**3**		1	26	172	90	30.42	3
**4**		1	36	170	73	25.25	2
**5**		0	36	168	75	26.57	4
**6**		1	30	178	102	32.19	3
**7**		1	34	180	90	27.77	3
**370**		0	29	178	60	29.38	2
**371**		1	34	180	67	29.68	1
**372**		0	38	178	70	23.63	0
**373**		1	31	180	65	33.95	2
**374**		0	43	173	72.5	24.22	3

^1^ W_DISC_NOH is the number of antihypertensive drugs at discharge.

**Table 4:** Confusion matrix of classification results.

Real Situation	Prediction Results	
	Positive Class	Negative Class
positive class	*TP* ^1^	*FN* ^2^
negative class	*FP* ^3^	*TN* ^4^

^1^ TP is Ture Positive; ^2^ FN is False Negative; ^3^ FP is False Positive; ^4^ TN is True Negative.

**Table 5:** The number of optimal feature subsets for each classifier under three criteria.

	Classifier	SVM	C4.5	RF	XGBoost
Criterion					
ACC (%)		3	2	3	4
F1 Measure		16	2	6	3
AUC		9	2	3	9

**Table 6:** Prediction performance (accuracy, F1 measure, and *AUC*) of each classifier using their optimal features subset.

	Classifier	SVM	C4.5	RF	XGBoost
Criterion					
ACC (%)		75.80%	86.30%	88.98%	94.36%
F1 Measure		0.626	0.819	0.859	0.875
AUC		0.660	0.839	0.871	0.927

## Appendix A

Table A1 is an explanation of the abbreviation in Table 1.

**Table A1:** The explanation of the abbreviated in Table 1.

NO.	Abbreviations	Explanation
1	SEX	sex
2	AGE	age
3	HEIGHT	height
4	WEIGHT	weight
5	BMI	body mass index
6	HR	heart rate
7	PULSE	pulse
8	RYSBPL	left arm systolic pressure
9	RYDBPL	left arm diastolic pressure
10	HTBEGIN	initial hypertension age
11	ZGSBP	highest systolic blood pressure
12	ZGDBP	highest diastolic blood pressure
13	PSSBP1	normal systolic blood pressure
14	PSDBP1	normal diastolic blood pressure
15	AO	ascending aorta diameter
16	LA	left atrium
17	IVSD	ventricular septal thickness
18	LV	left ventricular end diastolic diameter
19	EF	ejection fraction
20	LVPWd	thickness of the back wall
21	RVd	right ventricle
22	WBC	white blood cell
23	NEUT	percentage of neutrophils
24	RBC	red blood cells
25	HB	hemoglobin
26	PLT	platelet
27	UKET	ketone body
28	USG	specific gravity of urine
29	USG1	USG tube type
30	ALT	alanine aminotransferase
31	AST	aspartate aminotransferase
32	K	serum potassium
33	Na	serum sodium
34	Cl	serum chlorine
35	GLU	blood sugar
36	CREA	creatinine
37	BUN	urea nitrogen
38	URIC	uric acid
39	HSCRP	high-sensitivity C-reactive protein
40	TG	triglyceride
41	TC	triacylglycerol
42	HDLC	high density lipoprotein cholesterol
43	LDLC	low density lipoprotein cholesterol
44	FT3	serum free triiodothyronine
45	FT4	free thyroxine
46	T3	triiodothyronine
47	T4	tetraiodothyronine
48	TSH	thyroid stimulating hormone
49	MAUCR	urinary microalbumin/creatinine
50	HUPRO	4-hour urine protein quantitation
51	HBLAC	glycated hemoglobin
52	HCY	homocysteine
53	ESR	erythrocyte sedimentation rate
54	CRP	C-reactive protein
55	NTPRO	amino terminal precursor protein of brain natural peptide
56	ET	endothelin
57	RARMSBP	right upper limb systolic blood pressure
58	RARMDBP	right upper limb diastolic blood pressure
59	LARMSBP	left upper limb systolic blood pressure
60	LARMDBP	left upper limb diastolic blood pressure
61	RLEGSBP	right lower limb systolic blood pressure
62	RLEGDBP	right lower limb diastolic blood pressure
63	LLEGSBP	left lower extremity systolic blood pressure
64	LLEGDBP	left lower extremity diastolic blood pressure
65	BAPWVR	right brachium-ankle pulse wave conduction velocity
66	BAPWVL	left brachium-ankle pulse wave conduction velocity
67	ABIR	right ankle-brachium index
68	ABIL	left ankle-brachium index
69	AHI	hourly breathing number
70	APNEA	the longest apnea number
71	HYPOPNEA	the longest hypoventilation time
72	SAO2	the lowest SaO2%
73	MEANSAO2	the average SaO2%
74	MEANSBP	24h mean systolic blood pressure
75	MEANDBP	24h mean diastolic blood pressure
76	HIGHSBP	the highest systolic blood pressure
77	HIGHDBP	the highest diastolic blood pressure
78	LOWSBP	the lowest systolic blood pressure
79	LOWDBP	the lowest diastolic blood pressure
80	DAYMSBP	daytime average systolic blood pressure
81	DAYMDBP	daytime mean diastolic blood pressure
82	NIHTMSBP	nighttime average systolic blood pressure
83	NIHTMDBP	nighttime average diastolic blood pressure
84	W_DISC_NOHPT	number of antihypertensive drugs at discharge
